# (*E*)-4-{[2-(2-Furylcarbon­yl)hydrazinyl­idene]meth­yl}-2-meth­oxy­phenyl acetate

**DOI:** 10.1107/S1600536811008932

**Published:** 2011-03-15

**Authors:** Jun Xu, Xiao-yu Yue

**Affiliations:** a Department of Quality Detection and Management, Zhengzhou College of Animal Husbandry Engineering, Zhengzhou 450011, People’s Republic of China

## Abstract

The mol­ecule of the title Schiff base compound, C_15_H_14_N_2_O_5_, was obtained from a condensation reaction of 4-acet­oxy-3-meth­oxy­benzaldehyde and 2-furyl­carbonyl­hydrazide. In the mol­ecule, the furyl ring makes a dihedral angle of 14.63 (10)° with the benzene ring. In the crystal, inter­molecular N—H⋯O hydrogen bonds link the mol­ecules into chains along the *b* axis. Futhermore, weak C—H⋯O inter­actions connect the chains, forming corrugated layers parallel to (001). The dihedral angle between the rings is 14.63 (10)°.

## Related literature

Several phenyl­hydrazone derivatives have been shown to be potentially DNA-damaging and are mutagenic agents, see: Okabe *et al.* (1993 ▶). For bond lengths and angles in other hydrazone derivatives, see: Bakir & Gyles (2003 ▶); Baughman *et al.* (2004 ▶); Ohba (1996 ▶); Yao & Jing (2007 ▶).
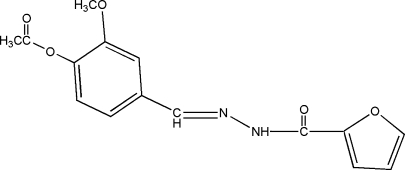


## Experimental

### 

#### Crystal data

C_15_H_14_N_2_O_5_
*M* *_r_* = 302.28
Orthorhombic, 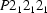

*a* = 4.9987 (2) Å
*b* = 13.4200 (5) Å
*c* = 21.5876 (8) Å
*V* = 1448.15 (10) Å^3^
*Z* = 4
Mo *K*α radiation
μ = 0.11 mm^−1^
*T* = 296 K
0.21 × 0.19 × 0.18 mm

#### Data collection

Bruker SMART CCD area-detector diffractometer
Absorption correction: multi-scan (*SADABS*; Bruker, 1998 ▶) *T*_min_ = 0.973, *T*_max_ = 0.977
39211 measured reflections
3626 independent reflections
2988 reflections with *I* > 2σ(*I*)
*R*_int_ = 0.037

#### Refinement

*R*[*F* ^2^ > 2σ(*F* ^2^)] = 0.037
*wR*(*F* ^2^) = 0.105
*S* = 1.04
3626 reflections
200 parameters
H-atom parameters constrained
Δρ_max_ = 0.17 e Å^−3^
Δρ_min_ = −0.12 e Å^−3^

### 

Data collection: *SMART* (Bruker, 1998 ▶); cell refinement: *SAINT* (Bruker, 1998 ▶); data reduction: *SAINT*; program(s) used to solve structure: *SHELXTL* (Sheldrick, 2008 ▶); program(s) used to refine structure: *SHELXTL*; molecular graphics: *XP* in *SHELXTL*, *ORTEPIII* (Burnett & Johnson, 1996 ▶) and *PLATON* (Spek, 2009 ▶); software used to prepare material for publication: *SHELXTL*.

## Supplementary Material

Crystal structure: contains datablocks global, I. DOI: 10.1107/S1600536811008932/dn2664sup1.cif

Structure factors: contains datablocks I. DOI: 10.1107/S1600536811008932/dn2664Isup2.hkl

Additional supplementary materials:  crystallographic information; 3D view; checkCIF report

## Figures and Tables

**Table 1:** Hydrogen-bond geometry (Å, °)

*D*—H⋯*A*	*D*—H	H⋯*A*	*D*⋯*A*	*D*—H⋯*A*
N1—H1*A*⋯O2^i^	0.86	2.25	2.9414 (18)	137
C1—H1*B*⋯O2^ii^	0.93	2.38	3.217 (2)	149

Symmetry codes: (i) 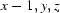; (ii) 
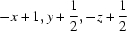
.

**Fig. 1.**
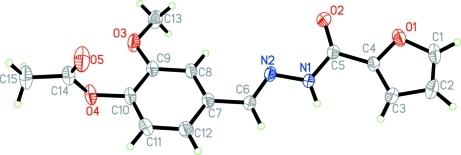
Molecular view of (I) with the atom labeling scheme. Displacement ellipsoids are drawn at the 30% probability level. H atoms are represented as small spheres of arbitrary radii.

**Fig. 2.**
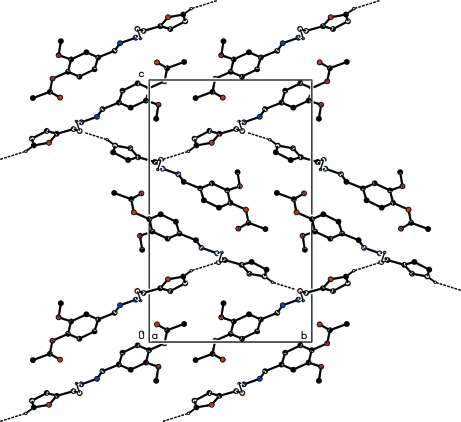
Packing view of (I) projected down the a axis. Hydrogen bonds are shown as dashed lines. H atoms not involved in hydrogen bondings have been omitted for clarity.

## supplementary crystallographic information

### Comment

Several phenylhydrazone derivatives have been shown to be potentially DNA-damaging and are mutagenic agents (Okabe *et al.* 1993). As part of our ongoing studies of Schiff bases, in this paper, we have synthesized the title compound and report the crystal structure.

The molecule adopts an E geometry with respect to the C=N bond (Fig. 1). The molecule is not planar, the dihedral angle between the furyl ring and the benzene ring is 14.63 (10)°, Bond lengths and bond angles agree with those of other hydrazone derivatives (Ohba, 1996; Baughman *et al.*, 2004; Yao & Jing *et al.*, 2007; Bakir & Gyles, 2003)

In the Crystal, Intermolecular N—H···O hydrogen bond link the molecules to form chains along the b axis. The chains are further connected to form corrugated layers parallel to the (0 0 1) plane (Table 1, Fig. 2).

### Experimental

Furan-2-carbohydrazine (1 mmol, 0.126 g) was dissolved in anhydrous ethanol (10 ml), The mixture was stirred for several minitutes at 351k, 4-acetoxy-3-methoxybenzaldehyde (1 mmol, 0.194 g) in ethanol (10 mm l) was added dropwise and the mixture was stirred at refluxing temperature for 3 h. The product was isolated and recrystallized from DMF, single crystals of (I) was obtained after one month.

### Refinement

All H atoms were positioned geometrically and refined as riding with C—H=0.93 (aromatic), 0.97(methylene), 0.96 Å(methyl) and N—H=0.86 Å, with *U*_iso_(H)=1.2*U*_eq_(CH, CH2 or NH) and *U*_iso_(H)=1.5*U*_eq_(C).

In the absence of significant anomalous scattering, the absolute structure could not be reliably determined and then any references to the Flack parameter were removed.

### Crystal data

**Table:**

C_15_H_14_N_2_O_5_	*F*(000) = 632
*M**_r_* = 302.28	*D*_x_ = 1.387 Mg m^−^^3^
Orthorhombic, *P*2_1_2_1_2_1_	Mo *K*α radiation, λ = 0.71073 Å
Hall symbol: P 2ac 2ab	Cell parameters from 2890 reflections
*a* = 4.9987 (2) Å	θ = 2.8–25.0°
*b* = 13.4200 (5) Å	µ = 0.11 mm^−^^1^
*c* = 21.5876 (8) Å	*T* = 296 K
*V* = 1448.15 (10) Å^3^	Block, colorless
*Z* = 4	0.21 × 0.19 × 0.18 mm

### Data collection

**Table:**

Bruker SMART CCD area-detector diffractometer	3626 independent reflections
Radiation source: fine-focus sealed tube	2988 reflections with *I* > 2σ(*I*)
graphite	*R*_int_ = 0.037
ω scans	θ_max_ = 28.4°, θ_min_ = 1.8°
Absorption correction: multi-scan (*SADABS*; Bruker, 1998)	*h* = −6→6
*T*_min_ = 0.973, *T*_max_ = 0.977	*k* = −17→17
39211 measured reflections	*l* = −28→28

### Refinement

**Table:**

Refinement on *F*^2^	Secondary atom site location: difference Fourier map
Least-squares matrix: full	Hydrogen site location: inferred from neighbouring sites
*R*[*F*^2^ > 2σ(*F*^2^)] = 0.037	H-atom parameters constrained
*wR*(*F*^2^) = 0.105	*w* = 1/[σ^2^(*F*_o_^2^) + (0.0588*P*)^2^ + 0.1124*P*] where *P* = (*F*_o_^2^ + 2*F*_c_^2^)/3
*S* = 1.04	(Δ/σ)_max_ = 0.001
3626 reflections	Δρ_max_ = 0.17 e Å^−^^3^
200 parameters	Δρ_min_ = −0.12 e Å^−^^3^
0 restraints	Extinction correction: *SHELXTL* (Sheldrick, 2008), Fc^*^=kFc[1+0.001xFc^2^λ^3^/sin(2θ)]^-1/4^
Primary atom site location: structure-invariant direct methods	Extinction coefficient: 0.014 (2)

### Special details

**Table:**

Geometry. All e.s.d.'s (except the e.s.d. in the dihedral angle between two l.s. planes) are estimated using the full covariance matrix. The cell e.s.d.'s are taken into account individually in the estimation of e.s.d.'s in distances, angles and torsion angles; correlations between e.s.d.'s in cell parameters are only used when they are defined by crystal symmetry. An approximate (isotropic) treatment of cell e.s.d.'s is used for estimating e.s.d.'s involving l.s. planes.
Refinement. Refinement of *F*^2^ against ALL reflections. The weighted *R*-factor *wR* and goodness of fit *S* are based on *F*^2^, conventional *R*-factors *R* are based on *F*, with *F* set to zero for negative *F*^2^. The threshold expression of *F*^2^ > σ(*F*^2^) is used only for calculating *R*-factors(gt) *etc*. and is not relevant to the choice of reflections for refinement. *R*-factors based on *F*^2^ are statistically about twice as large as those based on *F*, and *R*- factors based on ALL data will be even larger.

### Fractional atomic coordinates and isotropic or equivalent isotropic displacement parameters (Å^2^)

**Table:**

	*x*	*y*	*z*	*U*_iso_*/*U*_eq_	
N2	0.1299 (3)	0.82093 (8)	0.14030 (6)	0.0421 (3)	
N1	0.1018 (3)	0.91747 (9)	0.16153 (6)	0.0457 (3)	
H1A	−0.0498	0.9473	0.1583	0.055*	
O4	−0.0888 (3)	0.40544 (8)	0.00497 (5)	0.0589 (3)	
O1	0.4391 (3)	1.11568 (9)	0.23940 (6)	0.0614 (3)	
O3	0.2649 (3)	0.44319 (8)	0.09449 (6)	0.0607 (3)	
O2	0.5320 (2)	0.92740 (9)	0.19504 (6)	0.0548 (3)	
C5	0.3114 (3)	0.96497 (10)	0.18735 (7)	0.0399 (3)	
C9	0.1023 (4)	0.51982 (10)	0.07856 (7)	0.0452 (3)	
C8	0.1028 (3)	0.61391 (10)	0.10565 (7)	0.0433 (3)	
H8A	0.2245	0.6285	0.1370	0.052*	
C6	−0.0733 (3)	0.78742 (11)	0.11141 (7)	0.0432 (3)	
H6A	−0.2222	0.8282	0.1063	0.052*	
C7	−0.0777 (3)	0.68616 (10)	0.08613 (7)	0.0420 (3)	
C10	−0.0806 (4)	0.50087 (11)	0.03118 (7)	0.0474 (4)	
C4	0.2508 (3)	1.06823 (10)	0.20534 (7)	0.0428 (3)	
O5	0.2054 (4)	0.45208 (11)	−0.06712 (7)	0.0836 (5)	
C12	−0.2646 (4)	0.66377 (12)	0.04072 (8)	0.0505 (4)	
H12A	−0.3910	0.7111	0.0291	0.061*	
C11	−0.2631 (4)	0.57083 (12)	0.01263 (7)	0.0525 (4)	
H11A	−0.3851	0.5561	−0.0186	0.063*	
C3	0.0474 (4)	1.12983 (14)	0.19440 (10)	0.0670 (5)	
H3A	−0.1064	1.1157	0.1718	0.080*	
C14	0.0652 (4)	0.38936 (12)	−0.04507 (8)	0.0552 (4)	
C2	0.1111 (5)	1.22134 (14)	0.22389 (11)	0.0748 (6)	
H2B	0.0064	1.2786	0.2247	0.090*	
C15	0.0306 (6)	0.28530 (14)	−0.06886 (9)	0.0775 (6)	
H15A	0.1423	0.2757	−0.1045	0.116*	
H15B	−0.1531	0.2749	−0.0801	0.116*	
H15C	0.0803	0.2387	−0.0372	0.116*	
C13	0.4284 (5)	0.45684 (14)	0.14794 (9)	0.0655 (5)	
H13A	0.5339	0.3981	0.1547	0.098*	
H13B	0.3168	0.4687	0.1834	0.098*	
H13C	0.5444	0.5129	0.1417	0.098*	
C1	0.3456 (5)	1.20943 (13)	0.24976 (9)	0.0657 (5)	
H1B	0.4358	1.2582	0.2721	0.079*	

### Atomic displacement parameters (Å^2^)

**Table:**

	*U*^11^	*U*^22^	*U*^33^	*U*^12^	*U*^13^	*U*^23^
N2	0.0475 (7)	0.0290 (5)	0.0499 (6)	0.0012 (5)	0.0038 (5)	−0.0045 (5)
N1	0.0406 (6)	0.0314 (6)	0.0650 (8)	0.0019 (5)	−0.0017 (6)	−0.0106 (6)
O4	0.0856 (9)	0.0378 (6)	0.0534 (6)	−0.0175 (6)	0.0034 (6)	−0.0124 (5)
O1	0.0652 (8)	0.0464 (6)	0.0725 (8)	−0.0082 (6)	−0.0158 (7)	−0.0114 (6)
O3	0.0807 (8)	0.0366 (5)	0.0648 (7)	0.0077 (6)	−0.0113 (7)	−0.0101 (5)
O2	0.0457 (6)	0.0441 (6)	0.0746 (8)	0.0061 (5)	−0.0080 (6)	−0.0052 (5)
C5	0.0438 (8)	0.0336 (7)	0.0424 (7)	0.0003 (6)	0.0013 (6)	−0.0016 (5)
C9	0.0554 (9)	0.0327 (7)	0.0474 (8)	−0.0036 (6)	0.0012 (7)	−0.0045 (6)
C8	0.0485 (8)	0.0353 (7)	0.0460 (7)	−0.0037 (6)	−0.0013 (6)	−0.0068 (6)
C6	0.0444 (7)	0.0354 (7)	0.0498 (8)	0.0016 (6)	0.0015 (7)	−0.0050 (6)
C7	0.0469 (8)	0.0353 (7)	0.0439 (7)	−0.0050 (6)	0.0033 (6)	−0.0034 (6)
C10	0.0622 (10)	0.0349 (7)	0.0451 (8)	−0.0106 (7)	0.0036 (7)	−0.0079 (6)
C4	0.0462 (8)	0.0349 (7)	0.0475 (8)	−0.0037 (6)	−0.0005 (6)	−0.0056 (6)
O5	0.1165 (13)	0.0615 (8)	0.0730 (9)	−0.0209 (9)	0.0306 (9)	−0.0194 (7)
C12	0.0532 (9)	0.0444 (8)	0.0539 (9)	−0.0024 (7)	−0.0056 (7)	−0.0015 (7)
C11	0.0616 (10)	0.0474 (8)	0.0483 (8)	−0.0122 (8)	−0.0087 (7)	−0.0041 (7)
C3	0.0597 (10)	0.0470 (9)	0.0944 (14)	0.0113 (8)	−0.0142 (11)	−0.0217 (9)
C14	0.0775 (11)	0.0424 (8)	0.0457 (8)	−0.0024 (8)	−0.0066 (8)	−0.0082 (7)
C2	0.0847 (15)	0.0432 (9)	0.0965 (15)	0.0123 (10)	0.0050 (13)	−0.0206 (10)
C15	0.1201 (18)	0.0464 (9)	0.0660 (11)	−0.0009 (11)	−0.0085 (12)	−0.0196 (9)
C13	0.0848 (13)	0.0467 (9)	0.0648 (11)	0.0133 (9)	−0.0169 (10)	−0.0028 (8)
C1	0.0883 (14)	0.0406 (9)	0.0682 (11)	−0.0136 (9)	0.0057 (11)	−0.0161 (8)

### Geometric parameters (Å, °)

**Table:**

N2—C6	1.2742 (19)	C10—C11	1.369 (3)
N2—N1	1.3814 (16)	C4—C3	1.331 (2)
N1—C5	1.3471 (19)	O5—C14	1.194 (2)
N1—H1A	0.8600	C12—C11	1.387 (2)
O4—C14	1.344 (2)	C12—H12A	0.9300
O4—C10	1.4007 (17)	C11—H11A	0.9300
O1—C4	1.3537 (18)	C3—C2	1.419 (3)
O1—C1	1.361 (2)	C3—H3A	0.9300
O3—C9	1.355 (2)	C14—C15	1.498 (2)
O3—C13	1.426 (2)	C2—C1	1.308 (3)
O2—C5	1.2242 (18)	C2—H2B	0.9300
C5—C4	1.4706 (19)	C15—H15A	0.9600
C9—C8	1.3915 (19)	C15—H15B	0.9600
C9—C10	1.395 (2)	C15—H15C	0.9600
C8—C7	1.390 (2)	C13—H13A	0.9600
C8—H8A	0.9300	C13—H13B	0.9600
C6—C7	1.4644 (19)	C13—H13C	0.9600
C6—H6A	0.9300	C1—H1B	0.9300
C7—C12	1.387 (2)		
			
C6—N2—N1	114.35 (12)	C11—C12—H12A	120.0
C5—N1—N2	120.13 (12)	C7—C12—H12A	120.0
C5—N1—H1A	119.9	C10—C11—C12	119.52 (15)
N2—N1—H1A	119.9	C10—C11—H11A	120.2
C14—O4—C10	117.05 (13)	C12—C11—H11A	120.2
C4—O1—C1	106.58 (15)	C4—C3—C2	106.64 (17)
C9—O3—C13	116.85 (12)	C4—C3—H3A	126.7
O2—C5—N1	124.19 (13)	C2—C3—H3A	126.7
O2—C5—C4	122.54 (14)	O5—C14—O4	122.91 (15)
N1—C5—C4	113.27 (13)	O5—C14—C15	126.03 (19)
O3—C9—C8	125.51 (15)	O4—C14—C15	111.05 (17)
O3—C9—C10	116.14 (13)	C1—C2—C3	106.66 (18)
C8—C9—C10	118.34 (15)	C1—C2—H2B	126.7
C7—C8—C9	120.29 (15)	C3—C2—H2B	126.7
C7—C8—H8A	119.9	C14—C15—H15A	109.5
C9—C8—H8A	119.9	C14—C15—H15B	109.5
N2—C6—C7	121.46 (14)	H15A—C15—H15B	109.5
N2—C6—H6A	119.3	C14—C15—H15C	109.5
C7—C6—H6A	119.3	H15A—C15—H15C	109.5
C12—C7—C8	120.03 (13)	H15B—C15—H15C	109.5
C12—C7—C6	118.33 (14)	O3—C13—H13A	109.5
C8—C7—C6	121.63 (14)	O3—C13—H13B	109.5
C11—C10—C9	121.73 (13)	H13A—C13—H13B	109.5
C11—C10—O4	119.31 (15)	O3—C13—H13C	109.5
C9—C10—O4	118.82 (15)	H13A—C13—H13C	109.5
C3—C4—O1	109.59 (13)	H13B—C13—H13C	109.5
C3—C4—C5	134.06 (15)	C2—C1—O1	110.52 (17)
O1—C4—C5	116.33 (13)	C2—C1—H1B	124.7
C11—C12—C7	120.00 (16)	O1—C1—H1B	124.7

### Hydrogen-bond geometry (Å, °)

**Table:**

*D*—H···*A*	*D*—H	H···*A*	*D*···*A*	*D*—H···*A*
N1—H1A···O2^i^	0.86	2.25	2.9414 (18)	137
C1—H1B···O2^ii^	0.93	2.38	3.217 (2)	149

Symmetry codes: (i) *x*−1, *y*, *z*; (ii) −*x*+1, *y*+1/2, −*z*+1/2.
